# Classification and characterisation of extracellular vesicles‐related tuberculosis subgroups and immune cell profiles

**DOI:** 10.1111/jcmm.17836

**Published:** 2023-07-06

**Authors:** Peipei Zhou, Jie Shen, Xiao Ge, Fang Ding, Hong Zhang, Xinlin Huang, Chao Zhao, Meng Li, Zhenpeng Li

**Affiliations:** ^1^ School of Medical Laboratory Weifang Medical University Weifang China; ^2^ Respiratory Medicine Affiliated Hospital of Weifang Medical University Weifang China; ^3^ School of Public Health Weifang Medical University Weifang China; ^4^ Office of Academic Affairs Weifang Medical University Weifang China

**Keywords:** biomarkers, extracellular vesicles, immune cell profiles, single‐cell RNA sequence, subclusters, tuberculosis

## Abstract

Around the world, tuberculosis (TB) remains one of the most common causes of morbidity and mortality. The molecular mechanism of *Mycobacterium tuberculosis* (Mtb) infection is still unclear. Extracellular vesicles (EVs) play a key role in the onset and progression of many disease states and can serve as effective biomarkers or therapeutic targets for the identification and treatment of TB patients. We analysed the expression profile to better clarify the EVs characteristics of TB and explored potential diagnostic markers to distinguish TB from healthy control (HC). Twenty EVs‐related differentially expressed genes (DEGs) were identified, and 17 EVs‐related DEGs were up‐regulated and three DEGs were down‐regulated in TB samples, which were related to immune cells. Using machine learning, a nine EVs‐related gene signature was identified and two EVs‐related subclusters were defined. The single‐cell RNA sequence (scRNA‐seq) analysis further confirmed that these hub genes might play important roles in TB pathogenesis. The nine EVs‐related hub genes had excellent diagnostic values and accurately estimated TB progression. TB's high‐risk group had significantly enriched immune‐related pathways, and there were substantial variations in immunity across different groups. Furthermore, five potential drugs were predicted for TB using CMap database. Based on the EVs‐related gene signature, the TB risk model was established through a comprehensive analysis of different EV patterns, which can accurately predict TB. These genes could be used as novel biomarkers to distinguish TB from HC. These findings lay the foundation for further research and design of new therapeutic interventions aimed at treating this deadly infectious disease.

## 1 INTRODUCTION

Tuberculosis (TB) is a contagious disease caused by *Mycobacterium tuberculosis* (Mtb), which primarily affects the lungs.^1^ Globally, TB is the most widespread communicable disease in the world, causing the most deaths and illnesses. The emergence of multidrug‐resistant and universally drug‐resistant Mtb strains hampers global TB control.^2^ Prior to the Coronavirus (COVID‐19) pandemic, TB was the leading cause of infection‐related death before HIV/AIDS. The aetiology and pathogenesis of TB can therefore be used to guide clinical diagnosis and treatment, resulting in improved clinical outcomes.

Extracellular vesicles (EVs) are membrane‐derived lipid bilayers present in all three domains of life that package and produce cytoplasmic compounds such as nucleic acids and proteins.^3^ The size of EVs varies from 50 to 200 nanometres and they are secreted by a variety of mammalian cells^4^ and can deliver cargo to nearby cells or cells throughout the body.^3^ The molecular cargo portion of an EV reflects not only its original cell content but also changes in conditions (stress or disease).^5^ They are involved in maintaining normal physiology and possibly in various diseases.^6^, ^7^, ^8^, ^9^ EVs have become an important process in bacterial biology and host‐pathogen interactions. Secreted hydrophobic molecules, lipids, proteins and glycolipids encapsulated by EVs allow for long‐range interactions with the host, which have been implicated in the possible involvement of TB pathogenesis.^9^

In general, EVs regulation disorders tend to be associated with diseases because EVs have a wide range of functions in a variety of physiological processes. EVs play important roles in the pathogenesis of tuberculosis infection,^10^ including the inhibition of antigen presentation, immune inhibition, vaccination, Human TB biomarkers, immune cell recruitment, antigen presentation and macrophage activation. However, the comprehensive summary of the relationship between EVs and TB is not clear, and the detailed function research of EVs in TB is less. To explore the possible pathogenesis of TB, we analysed the expression differences between healthy control (HC) and TB samples using Expression Omnibus (GEO) database. Through machine learning, we identified a EVs‐related gene signature, and based on this, we divided TB patients into two groups with significantly different patterns, and analysed the differences in functions and immune cells between the two subclusters. So as to provide a novel molecular perspective on the pathogenesis of TB as well as to aid in diagnosing, prognosizing and treating the disease.

## 2 MATERIALS AND METHODS

### 2.1 Datasets collection

The RNA‐seq gene expression data samples used in this study were obtained from the GeneExpression Omnibus (GEO) (http://www.ncbi.nlm.nih.gov/geo/). The datasets with accession numbers GSE157657, GSE41055, GSE62525, GSE98461, GSE19444, GSE34608 and GSE83456. All data were converted to standard format for further analysis. The EVs‐related genes were obtained from a public database (GeneCard: https://www.genecards.org/).^11^ 1017 EVs‐related genes were involved in this study with a relevance score >5.0.

### 2.2 Identification of differentially expressed genes (DEGs)

We performed differential gene analysis using the R package ‘limma’,^12^ choosing a threshold of *p* < 0.05 and |log2 fold change| > 0.585 as thresholds to assign DEGs between TB and HC samples. Use volcano plots and heatmaps to visualize representation data for different genes.

### 2.3 Functional enrichment analysis

The ‘clusterProfiler’ package in R or DAVID (https://david.ncifcrf.gov/) was used to further examine the bioinformatics data and for Gene Ontology (GO) enrichment analysis and Kyoto Encyclopedia of Genes and Genomes (KEGG) pathway analysis.^13^ Gene set variation analysis (GSVA)^14^ was performed to analyse different biological functions with the ‘c2.cp.reactome.v7.5.1.symbols.gmt’, ‘c2.cp.kegg.symbols.gmt’ and ‘c5.go.symbols.gmt’ databases.

### 2.4 Evaluating the immune cell infiltration

The CIBERSORT algorithm was used to calculate the percentages of 22 immune cell types in TB patients, using a total of 547 characteristic genes (referred to as LM22 characteristic in CIBERSORT) to evaluate the infiltration level.^15^

### 2.5 Single‐cell data preprocessing, gene expression quantification and cell‐type identification

The raw sequencing reads obtained from NCBI Short Read Archive (SRA) with the accession numbers SRR11038989 and SRR11038994^16^ were processed by Cell Ranger (6.1.2) and aligned to the human reference genome (GRCh38). The ‘Seurat’ R package was then used to import the unique molecular identifier (UMI) count matrices. Cells with <200 or >3500 genes or a high mitochondrial transcript ratio (>0.07) were filtered out. For the remaining cells, we used the NormalizedData, Seurat indVariableFeatures, ScaleData, JackStraw and FindNeighbors functions in the Seurat package to process the data. tSNE was performed to reduce the dimensionality of the cells representing the cells. Clustering was then performed on the cells, and the clusters were annotated based on the marker gene composition from previous studies.^16^, ^17^

### 2.6 Machine learning

Extracellular vesicles‐related DEGs were identified by intersecting DEGs and EVs‐related genes. Four machine learning methods, including the least absolute shrinkage and selection operator (LASSO),^18^ Support vector machine (SVM),^19^ random forest (RF)^20^ and eXtreme Gradient Boosting (Xgboost)^21^ were employed to filter the potential TB genes, which were performed by using the ‘glmnet’, ‘kernlab’, ‘randomForest’ and ‘xgboost’ R packages. The purpose of using LASSO regression is to improve the predicted accuracy and comprehensibility of the model by means of regularisation. Furthermore, LASSO regression allows for the selection of relevant variables.^18^ SVM is an effective method that seeks to create a boundary between two classes, enabling the prediction of labels by utilizing single or multiple feature vectors.^19^ On the other hand, when it comes to forecasting continuous variables while minimizing variance, RF is a suitable technique due to its capability to achieve high precision, sensitivity and specificity. Moreover, RF does not impose restrictions on the conditions of variables.^20^ XGBoost is a widely‐used supervised machine learning algorithm that has notable scalability and user‐friendly functionalities, making it an ideal choice for model optimization and visualization.^21^ For RF and Xgboost analysis, the top 15 genes were selected for subsequent analysis. TB diagnosis relied on the intersection of genes. Moreover, the ‘circlize’ package^22^ was used to visualize the interaction between the EVs‐related hub genes. The ‘pROC’ R package was used to conduct a receiver operating characteristic (ROC) analysis in order to further assess the ability of the prediction model to differentiate between TB and HC.^23^

### 2.7 Unsupervised clustering of nine EVs‐related genes

A consensus cluster analysis was conducted using ‘ConsensusClusterPlus’ R package,^24^ which was based on mRNA expression data of nine EVs‐related signature genes. Select maxK 9 for the CC parameter, pam for the ClusterAlg and Euclidean for the distance.

### 2.8 The connectivity map (CMap) analysis

The CMap (https://clue.io/) was used to prioritize small molecules. The ‘query’ module of CMap via the L1000 platform was used to interrogate the DEGs from patients with high and low‐risk groups. The top 100 up‐ and down‐regulated genes based on the log2‐fold change were submitted to the query, and the normalized connectivity score (norm_cs) was obtained. The five drugs with the top five norm_cs were chosen as the target drugs.

### 2.9 Statistical analysis

Statistical analysis of all data was performed using R 4.2.2. The *t*‐test or Wilcox test was used to compare the differences between the two groups. Spearman correlation was used to investigate the correlation between the expression level of EVs‐related hub genes and immune cells. A *p*‐value or adjusted *p*‐value of less than 0.05 was considered statistically significant.

## 3 RESULTS

### 3.1 DEGs identification and functional pathway enrichment

**FIGURE 1:**
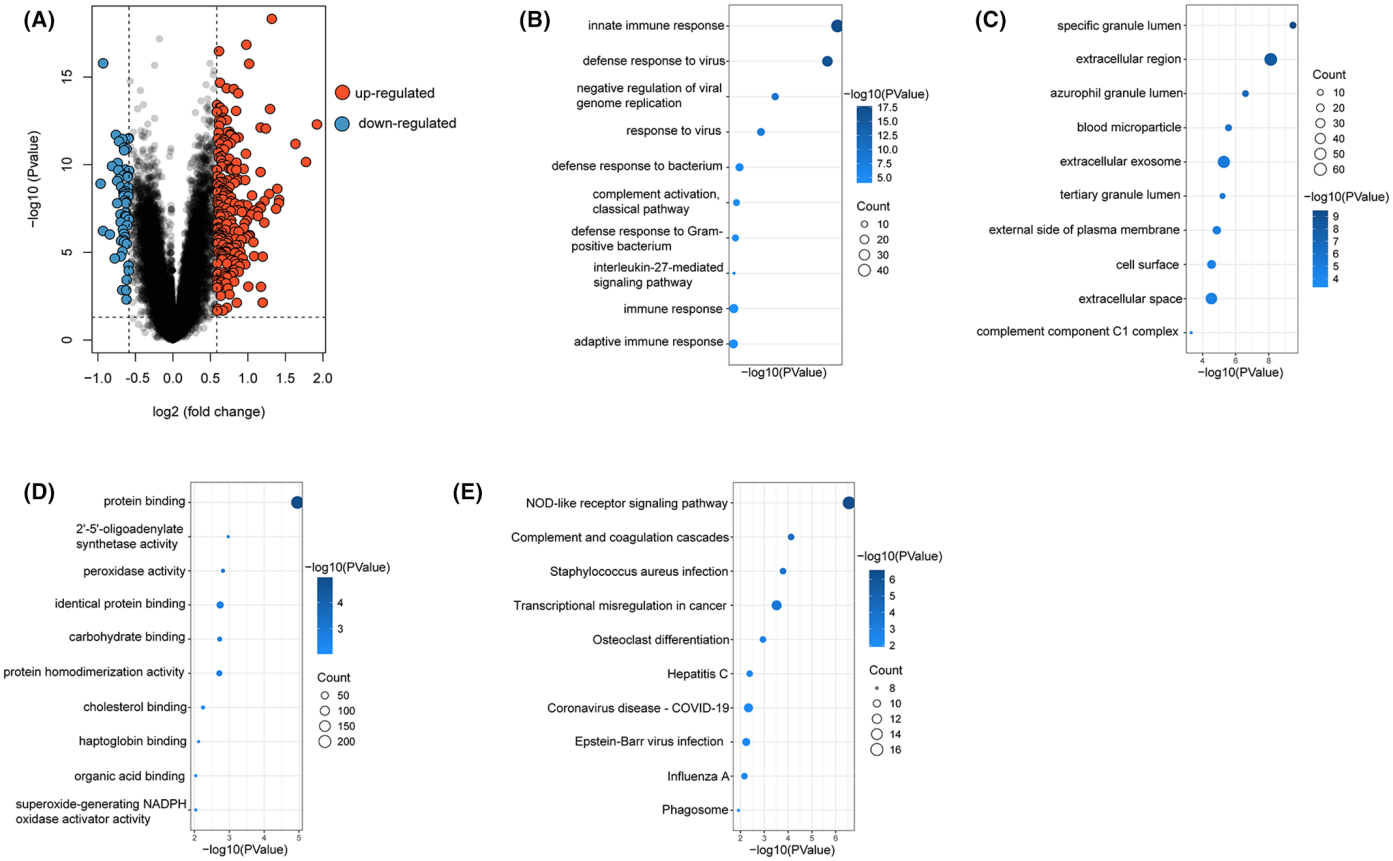
Differentially expressed genes and functional enrichment. (A) The volcano plotting of TB‐related DEGs. (B) The enrichment of GO BP. (C) The enrichment of GO CC. (D) The enrichment of GO MF. (E) The enrichment of KEGG analysis. GO, gene ontology; BP, biological process; CC, cellular component; MF, molecular function; KEGG, Kyoto Encyclopedia of Genes and Genomes.

The GSE157657 dataset was downloaded from the GEO database, which consisted of 38 HC samples and 565 TB samples. Gene expression patterns were distinct between the HC and TB samples (Figure 1A). We identified a total of 301 DEGs (242 of which were up‐regulated genes, 59 of which were down‐regulated genes). To explore the functions of the DEGs, GO and KEGG analyses were performed. GO biological processes (BP) analysis revealed that innate immune response, defence response to virus, negative regulation of viral genome replication, response to virus, defence response to bacterium, complement activation/classical pathway, defence response to Gram‐positive bacterium, interleukin‐27‐mediated signalling pathway, immune response and adaptive immune response were enriched (Figure 1B). Moreover, DEGs' most related cellular components (CC) were specific granule lumen, extracellular region, azurophil granule lumen, blood microparticle, etc. (Figure 1C). The molecular functions (MF) were enrichment of protein binding, 2′‐5′‐oligoadenylate synthetase activity, peroxidase activity, identical protein binding and carbohydrate binding (Figure 1D). The most DEGs‐related signalling pathway were shown in Figure 1E. These findings suggested that DEGs probably played an essential role in the regulation of the disease and immune response.

### 3.2 Evaluation of immune cell infiltration

**FIGURE 2:**
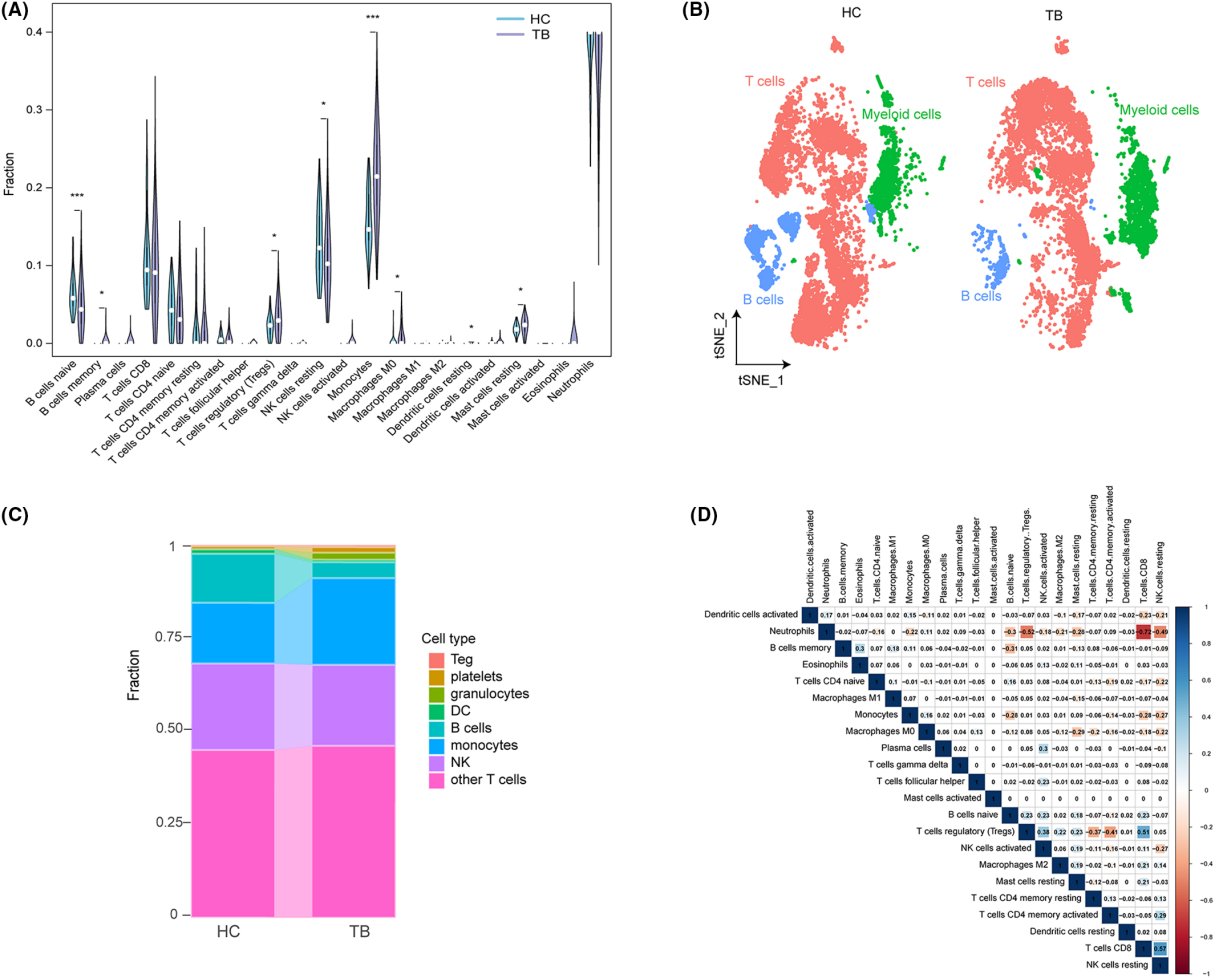
Immune cell infiltration in patients with TB. (A) The fraction of 22 types of immune cells. (B) scRNA analysis characterized major three groups. (C) The fraction of cells for different cell types between HC and TB. (D) Comparison of 22 immune cells between TB patients and controls. *p < 0.05; ***p < 0.001.

We found that these DEGs were involved in the immune response. Therefore, we infiltrated immune cells to further elucidate TB immune regulation. As shown in Figure 2A, TB samples had a higher proportion of memory B cells, regulatory T cells (Tregs), Monocytes, Macrophages M0, Dendritic cells (DCs) resting and resting mast cells. Meanwhile, TB samples had a lower proportion of naïve B cells and resting NK cells (Figure 2A).

We then carried out single‐cell RNA sequencing (scRNA‐seq) analysis on PBMCs derived from TB and HC (raw data obtained from NCBI). A total of 20,880 cells (10,373 from HC and 10,507 from TB) were taken into analysis. As shown in Figure 2B, Figures S1 and S2, three major clusters were identified by the LYZ, S100A9, S100A8, S100A12 and CD14 (myeloid cells makers), CD3D, CD3E, IL32 and CD2 (T cells makers) and CD79A, CD79B and MS4A1 (B cells makers). With the indicated makers, five subtypes (CD4^+^ T, CD8^+^ T, megakaryocyte‐like cells, Treg and NK), four subtypes (DC, platelets, monocytes and granulocytes) and three subtypes (follicular B cells, activated B cells and mature B cells) were determined in T cells cluster, myeloid cells cluster and B cells cluster, respectively (Figures S2–S4). Further analysis revealed that the fraction of different cells was consistent with our previous results (Figure 2C).

The association between 22 immune cell classes revealed that most immune cells, except activated mast cells, were closely related (Figure 2D). Different types of immune cells, especially innate immune cells, were uniquely infiltrated in TB patients, which might become the therapeutic targets of TB.

### 3.3 Identification of the EVs‐related gene signature

**FIGURE 3:**
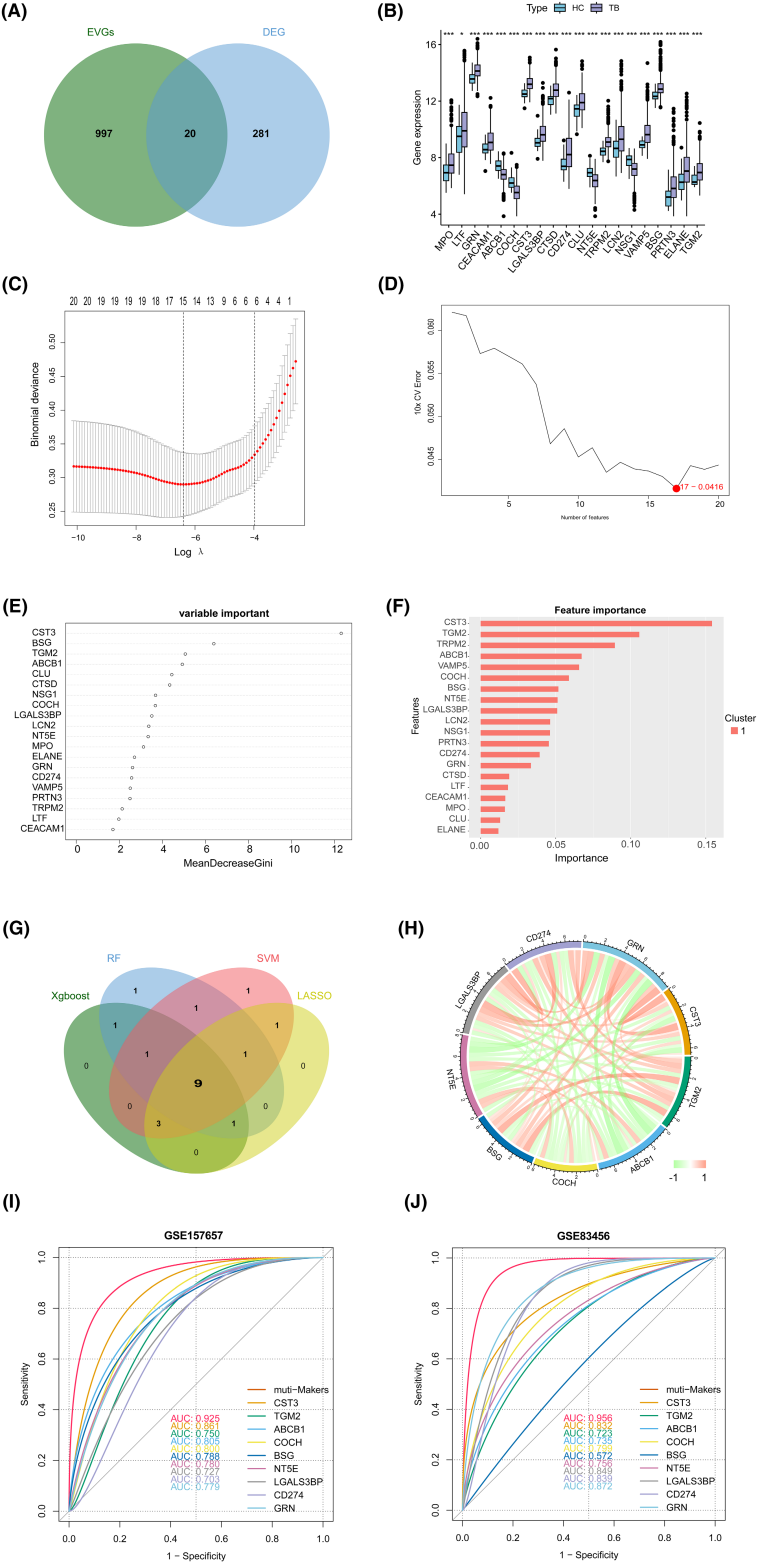
Screening of the EVs‐related gene signature. (A) The overlap of genes between EVs‐related genes and DEGs. (B) The expression of EVs‐related DEGs. (C‐F) Construction of EVs‐related gene signature using LASSO, SVM, RF and Xgboost. (G) The overlap of genes in four machine learning. (H) Circos plotting showing the relationship between the EVs‐related gene signature. (I, J) ROC curve of EVs‐related hub genes in TB diagnosis. DEGs, differentially expressed genes; LASSO, least absolute shrinkage and selection operator; RF, random forest; ROC, receiver operating characteristic; SVM, support vector machine; Xgboost, eXtreme Gradient Boosting. *p < 0.05; ***p < 0.001.

We identified 20 EVs‐related DEGs (MPO, LTF, GRN, CEACAM1, ABCB1, COCH, CST3, LGALS3BP, CTSD, CD274, CLU, NT5E, TRPM2, LCN2, NSG1, VAMP5, BSG, PRTN3, ELANE and TGM2) via intersection of the 1017 EVs‐related genes and 301 DEGs (Figure 3A). Except for ABCB1, COCH, NT5E and NSG1, most EVs‐related DEGs were highly expressed in TB samples (Figure 3B). Based on the 20 EVs‐related DEGs, four machine learning (LASSO, SVM, RF, Xgboost) identified nine EVs‐related hub genes, including CST3, TGM2, ABCB1, COCH, BSG, NT5E, LGALS3BP, CD274 and GRN (Figure 3C–G). The hub genes associated with EVs were found to be highly correlated with each other, as shown in Figure 3H.

Based on the EVs‐related gene signature, we performed ROC curves to predict TB. Notably, all the nine hub genes had a high AUC (Figure 3I). The results were further validated in the GEO database (GSE83456, GSE41055, GSE62525, GSE98461, GSE19444, GSE34608) (Figure 3J, Figure S5). These results indicated that all nine hub genes had excellent diagnostic values.

### 3.4 The relationship of EVs‐related gene signature with immune cells

**FIGURE 4:**
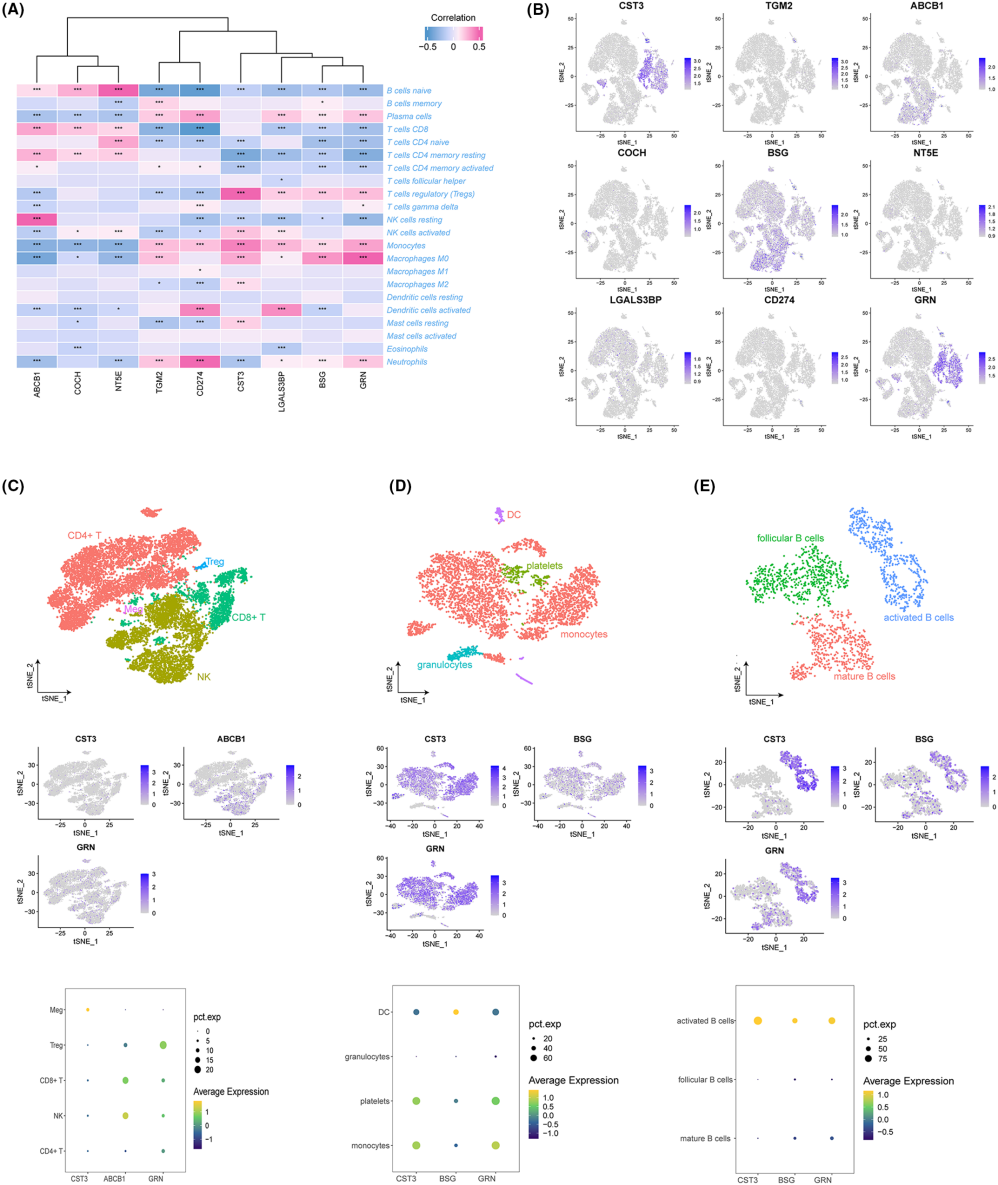
Correlation between immune cells with EVs‐related hub genes. (A) Heatmap showing the correlation between 22 immune cells with hub genes. (B) Expression of the EVs‐related hub genes in different cell groups. (C) Expression of CST3, ABCB1 and GRN in T Subtypes. (D) Expression of CST3, BSG and GRNA in myeloid Subtypes. (E) Expression of CST3, BSG and GRN in B Subtypes. *p < 0.05; ***p < 0.001.

We applied Spearman's correlation analysis to investigate the correlation between diagnostic genes and immune cell infiltration, in order to gain insights into the role of these genes in immune infiltration associated with EVs. Correlation analysis showed that nine EVs‐related signature genes exhibited a strong association with naïve B cells, plasma cells, CD8^+^ T cells, monocytes and neutrophils (Figure 4A). It was worth noting that the relationship between ABCB1, COCH, NT5T and immune cells was exactly opposite to the other six genes.

Subsequently, the expression levels and locations of the nine hub genes were determined by scRNA analysis. CST3, ABCB1, BSG and GRN had a high expression in different immune cells (Figure 4B). CST3 and GRN were dominantly expressed in B cells and Myeloid cells, while ABCB1 and BSG were enriched in T cells (Figure 4B). Further analysis showed that CST3 and GRN were dominantly expressed in all myeloid cell subtypes and activated B cells, while ABCB1 and BSG were dominantly enriched in NK cells and active B cells (Figure 4C‐E2).

Additionally, these genes were strongly enriched in immune‐related and infection pathways according to GSEA results (Figure S6).

### 3.5 EVs‐related gene signature‐based consensus clustering analyses

**FIGURE 5:**
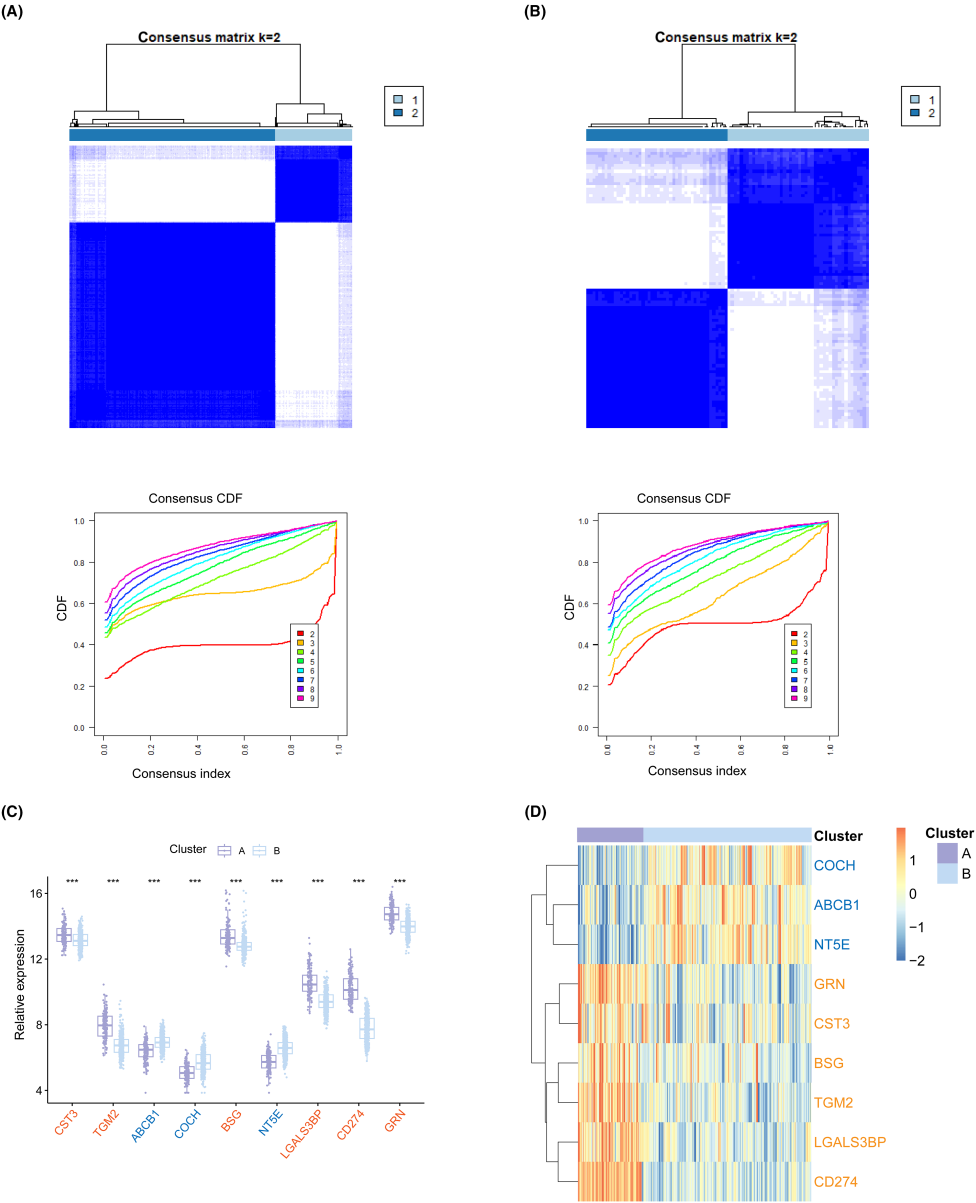
Identification of EVs‐related subtypes in tuberculosis. Subclusters identified with the EVs‐related hub genes in GSE157657 dataset (A) and GSE83456 (B). Boxplot (C) and Heatmap (D) showing the expression of EVs‐related hub genes between subtypes. CDF, cumulative distribution function; EVs, extracellular vesicles.

Consensus clustering of these nine hub EVs‐related genes was performed to identify novel subgroups of TB patients. In TB samples, k‐value 2 effectively separated them into two distinct clusters, indicating significant gene expression differences between the two groups (Figure 5A). Similar findings were also evident in the GSE83456 dataset (Figure 5B). Our results indicated that TB high‐risk group (cluster A) was associated with high expression levels of CST3, TGM2, BSG, LGALS3BP, CD274 and MPO, and low expression levels of ABCB1, COCH and NT5E (Figure 5C,D).

**FIGURE 6:**
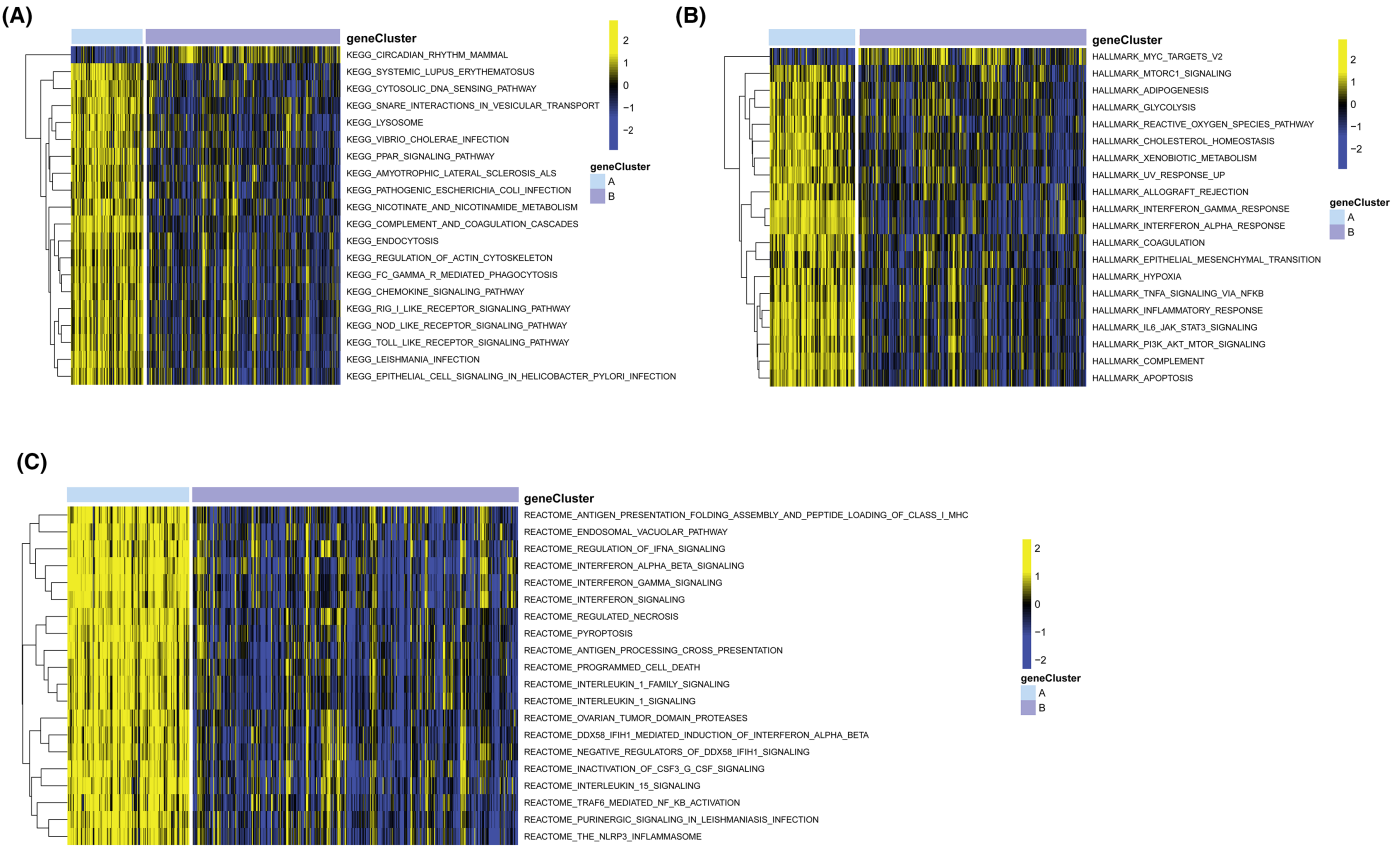
GSVA showing between EVs‐related subtypes. (A) Enrichment based on the KEGG pathway. (B) Enrichment based on the HALLMARK pathway. (C) Enrichment based on the Reactome pathway. GSVA, gene set variation analysis.

We found several pathways with differential expression through GSVA analysis and displayed them in a heatmap. Compared with cluster A, the KEGG pathways linked with circadian rhythm mammal were significantly enriched, while the expression of some immune pathways, such as chemokine signalling pathway, RIG I‐like receptor signalling pathway, NOD‐like receptor signalling pathway, Toll‐like receptor signalling pathway, were dramatically reduced (Figure 6A). Hallmark activities of MYC targets v2 were lower in cluster A, while IL6_JAK_STAT3_signalling, interferon gamma response, inflammatory response, etc. were higher (Figure 6B). The results of Reactome pathway showed that antigen presentation folding assembly and peptide loading of class I MHC, endosomal vacuolar pathway, regulation of IFNA signalling, interferon alpha beta signalling, interferon gamma signalling, etc. were enriched in cluster A (Figure 6C). It was evident from all of these results that the immune‐related pathways in high‐risk group (cluster A) were elevated and relative to those in low‐risk group (cluster B).

### 3.6 Subclusters related to EVs and functional distinctions

**FIGURE 7:**
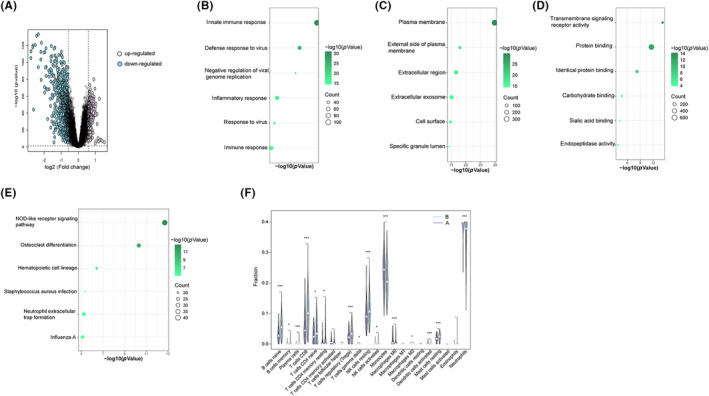
Functional enrichment analysis and immune cell infiltration between EVs‐related subtypes. (A) Volcano plotting of DEGs. (B–E) Enrichment of GO BP, CC, MF analysis. (F) The correlation between 22 immune cells in subtypes. *p < 0.05; ***p < 0.001.

To identify functional differences between the two subclusters, we performed a representational analysis of the differences. A total of 1053 DEGs (242 up‐regulated genes, 811 down‐regulated genes) were obtained (Figure 7A). Based on these DEGs, GO and KEGG analyses were conducted. GO BP analysis revealed that innate immune response, defence response to virus, negative regulation of viral genome replication, inflammatory response, immune response, etc. were enriched (Figure 7B). DEGs most related CC were plasma membrane, external side of plasma membrane, extracellular region, etc. (Figure 7C). The MF was enriched with transmembrane signalling receptor activity, protein binding, identical protein binding, etc. (Figure 7D). KEGG enrichment analysis revealed that several of these genes were enriched in the NOD‐like receptor signalling pathway, Neutrophil extracellular trap formation and other immune system compartments (Figure 7E).

Additionally, we performed an immune cell infiltration analysis using the subgroups. Consistent with the previous analysis, the proportion of innate immune cells (monocytes, Macrophages M0 and Dendritic cells activated) in high‐risk group A was higher, whereas the proportions of naïve B cells, CD8^+^ T cells, resting NK cells, etc. were lower than that in group B (Figure 7F).

### 3.7 Efficacy of EVs signature in predicting drug sensitivity

A CMap analysis was conducted to predict potential anti‐disease small molecule compounds for high‐risk patients. We screened five target drugs (pazopanib, baricitinib, BRD‐K09991945, pranlukast, masitinib) with the top five norm_cs (Table S1). The results showed that these drugs might be able to intervene in TB progression.

## 4 DISCUSSION

Infectious diseases such as TB are among the deadliest worldwide, with higher morbidity and mortality rates.^25^ There is, however, a lack of understanding regarding the molecular factors that can contribute to negative TB patient outcomes. TB is one of the most difficult infections to treat, and it takes several months of multi‐drug treatment to achieve a lasting cure.^26^ The reasons for requiring long‐term treatment are complex and multifactorial. Antibiotic resistance is a major challenge in the treatment of TB, and the COVID‐19 pandemic is further exacerbating the diagnosis, treatment and burden of TB.^27^, ^28^, ^29^ Although many patients with various TB have benefited from antibiotic therapy and others, the clinical effect of these therapies in an increasing number of patients is still disappointing.^30^, ^31^ Therefore, elucidating the molecular mechanism of TB pathogenesis and developing new therapies utilising these mechanisms are crucial for clinical treatment and success.

Over the past decade, the research field of EVs has developed rapidly, transitioning from fundamental biology to an area of significant clinical importance, which has begun to recognize the potential of harnessing EVs for the diagnosis and treatment of diseases.^32^ Additionally, EVs have emerged as potential tools for the diagnosis of tuberculosis, serving as biomarkers in the transition from faeces‐based diagnosis to more easily accessible biological fluids such as blood.^10^ According to our knowledge, this study is the first to analyse nine diverse and comprehensive EVs patterns in TB samples and identify two distinct EVs clusters, and further verify their excellent performance in other external queues as well. In our study, a total of 301 DEGs were determined between HC and TB patients. The GO and KEGG enrichment analysis revealed enrichment of immune response, which played an important role in TB occurrence and progression. Immune cell infiltration analysis demonstrated that memory B cells, Monocytes, Macrophages M0 and mast cells resting had a higher proportion in TB samples, which was consistent with previous studies.^33^, ^34^, ^35^, ^36^ This was further validated by scRNA analysis. Functional heterogeneity of memory B cell subsets may positively and negatively affect host immunity against TB.^37^ Tregs, a specialized subset of T cells, play an important role in preventing the development of autoimmune diseases by suppressing harmful immune cell activity.^38^ Monocytes are the major innate immune cells at the onset of Mtb infection and protect the host from intracellular pathogens.^39^, ^40^ Monocyte heterogeneity and differentiation into monocyte‐derived dendritic cells or monocyte‐derived macrophages have established a link between innate and adaptive immune responses.^41^ Mast cells play a critical role in the establishment and perpetuation of the inflammatory response leading to granuloma generation during tuberculosis.^35^ All of these provided further evidence of the importance of immunity in the development of TB.

Extracellular vesicles are essential to the development and function of biological process, and the dysfunction of EVs are closely related to many diseases. Hosts and EVs have been studied in the context of Mtb infection and disease. EVs can carry molecules, such as proteins, lipids, DNA and mRNA, and have been used as potential biomarkers for TB.^42^, ^43^, ^44^, ^45^, ^46^ However, the relevance of EVs‐related genes in TB has not been confirmed and further studies are needed. Through four machine learning algorithms, we finally obtained nine hub genes (CST3, TGM2, BSG, LGALS3BP, CD274, MPO, ABCB1, COCH and NT5E). In order to further clarify the relationship between these genes and TB, we then determined the correlation between them. It was found that EVs‐related genes interacted synergistically or antagonistically with TB patients. In addition, the hub EVs‐related genes were validated for identifying TB's high‐risk group, which showed good discrimination. BSG acts as a multifunctional glycoprotein by participating in multiple regulatory pathways related to proliferation, angiogenesis, ion transport or drug efflux, cell adhesion and migration, which is correlated with many diseases.^47^ By regulating the immune system, LGALS3BP can provide host protection during the development of diseases.^48^, ^49^ The role of CD274 on T cells and, to a lesser extent, dendritic cells in the chronic infectious disease of TB is well studied.^50^, ^51^ MPO induces necrosis in human neutrophils, promotes default apoptosis and subsequent phagocytosis of mycobacteria‐infected neutrophils by macrophages to control mycobacteria.^52^ It was known that CST3, TGM2, ABCB1 and COCH are biomarkers in the development of TB.^53^, ^54^, ^55^, ^56^ Further analysis of scRNA showed that CST3, ABCB1, BSG and GRN were highly expressed in different immune cells and the remaining five hub genes might regulate the immune process in different ways. However, the association of these genes in TB has not been documented and more research is required.

Using unsupervised cluster analysis, we identified two clusters associated with EVs‐related genes based on nine hub genes. GSVA results revealed that the immune‐related pathways exhibited elevated in TB high‐risk group. KEGG analysis also showed that DEGs were enriched in NOD‐like receptor signalling pathway, neutrophil extracellular trap formation, necroptosis, etc., which played a role in TB.^52^, ^56^, ^57^, ^58^, ^59^ The results of immune cell infiltration were consistent with our previous results and reflected its key role in TB immune process.

Further, we used CMap database to predict small‐molecular drugs based on EVs to treat TB, providing a certain basis for the treatment of TB. Therefore, future research could focus on the comprehensive mechanism of the expression of various EVs and their functional roles in the progression of TB to develop treatment strategies for EVs.

This study also has several limitations. First of all, these findings are based on extensive bioinformatics analysis and have not been experimentally or clinically validated. Our results need further confirmation. In addition, our data comes from the public database. As the original sequencing data is not available, there is a possibility of selection bias. In addition, our results need further verification since our sample size is still relatively small. Further research is needed to clarify the basic mechanism.

## 5 CONCLUSION

In conclusion, the EVs‐related gene signature suggested in this study can play a significant role in determining clinical outcomes for TB patients. And in‐depth study of EVs in TB will help to better understand the pathogenesis of TB and its potential as a biomarker and treatment target, and promote the development of effective treatment strategies.

## AUTHOR CONTRIBUTIONS

**Peipei Zhou:** Conceptualization (lead); methodology (equal); software (equal). **Jie Shen:** Methodology (equal); software (equal); writing – original draft (lead). **Xiao Ge:** Methodology (equal); software (supporting); writing – original draft (supporting). **Fang Ding:** Data curation (lead); visualization (lead). **Hong Zhang:** Writing – review and editing (equal). **Xinlin Huang:** Investigation (lead). **Chao Zhao:** Writing – review and editing (equal). **Meng Li:** Supervision (equal); writing – review and editing (equal). **Zhenpeng Li:** Funding acquisition (lead); supervision (equal); writing – review and editing (equal).

## FUNDING INFORMATION

This work was supported by Health Science and Technology Development Program of Shandong Province (grant numbers 202101060342), National Natural Science Foundation of China (grant numbers 32200512) and public‐sponsored domestic visiting program of Weifang Medical University (grant numbers 20237‐02; 20217‐20).

## CONFLICT OF INTEREST STATEMENT

The authors report there are no competing interests to declare.

## Supporting information


Figures S1–S6 and Table S1

## Data Availability

The authors confirm that the data supporting the findings of this study are available within the article and its supplementary materials. Further inquiries can be directed to the corresponding author.
