# Supplementary material for: Classification and characterisation of extracellular vesicles‐related tuberculosis subgroups and immune cell profiles
Source: J Cell Mol Med. 2023 Jul 6;27(17):2482–94. doi: 10.1111/jcmm.17836 (PMC10468662; doi:10.1111/jcmm.17836)
Supplement: Supplementary file 1 — Figures S1–S6 and Table S1 [file JCMM-27-2482-s001.docx]

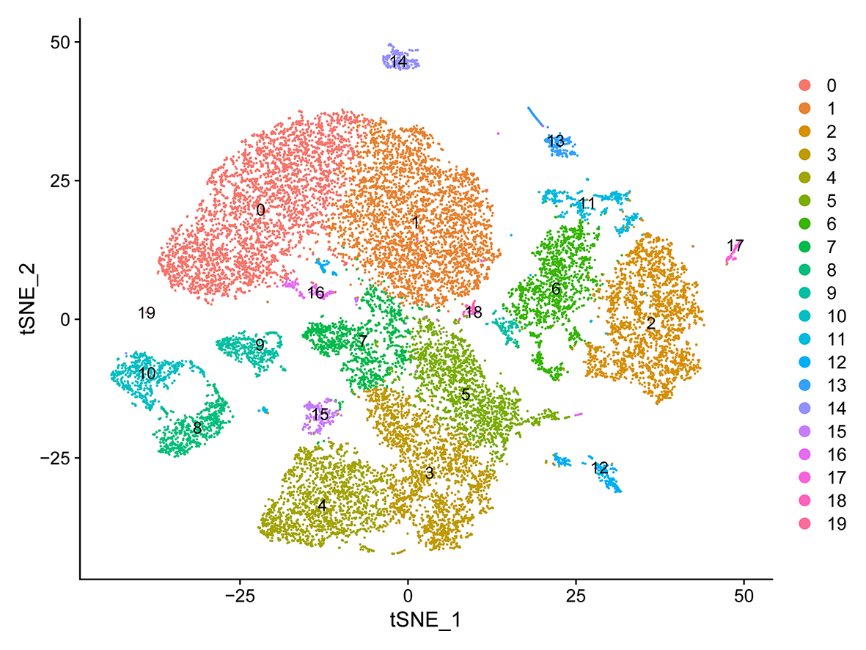


Figure S1 Subtypes of PBMC.


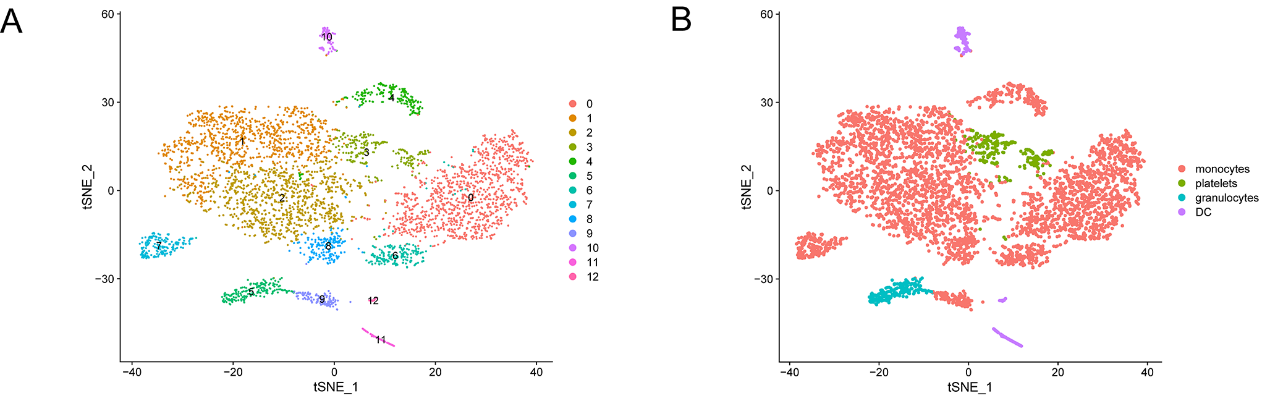


Figure S2 (A) Subtypes of myeloid cells. (B) Four subtypes in myeloid cells.


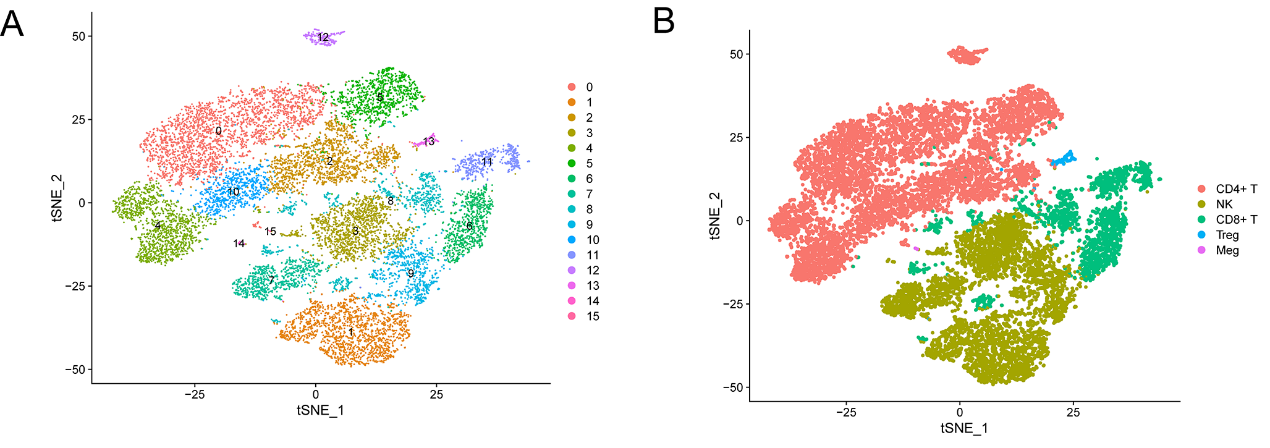


Figure S3 (A) Subtypes of T cells. (B) Five subtypes in T cells.


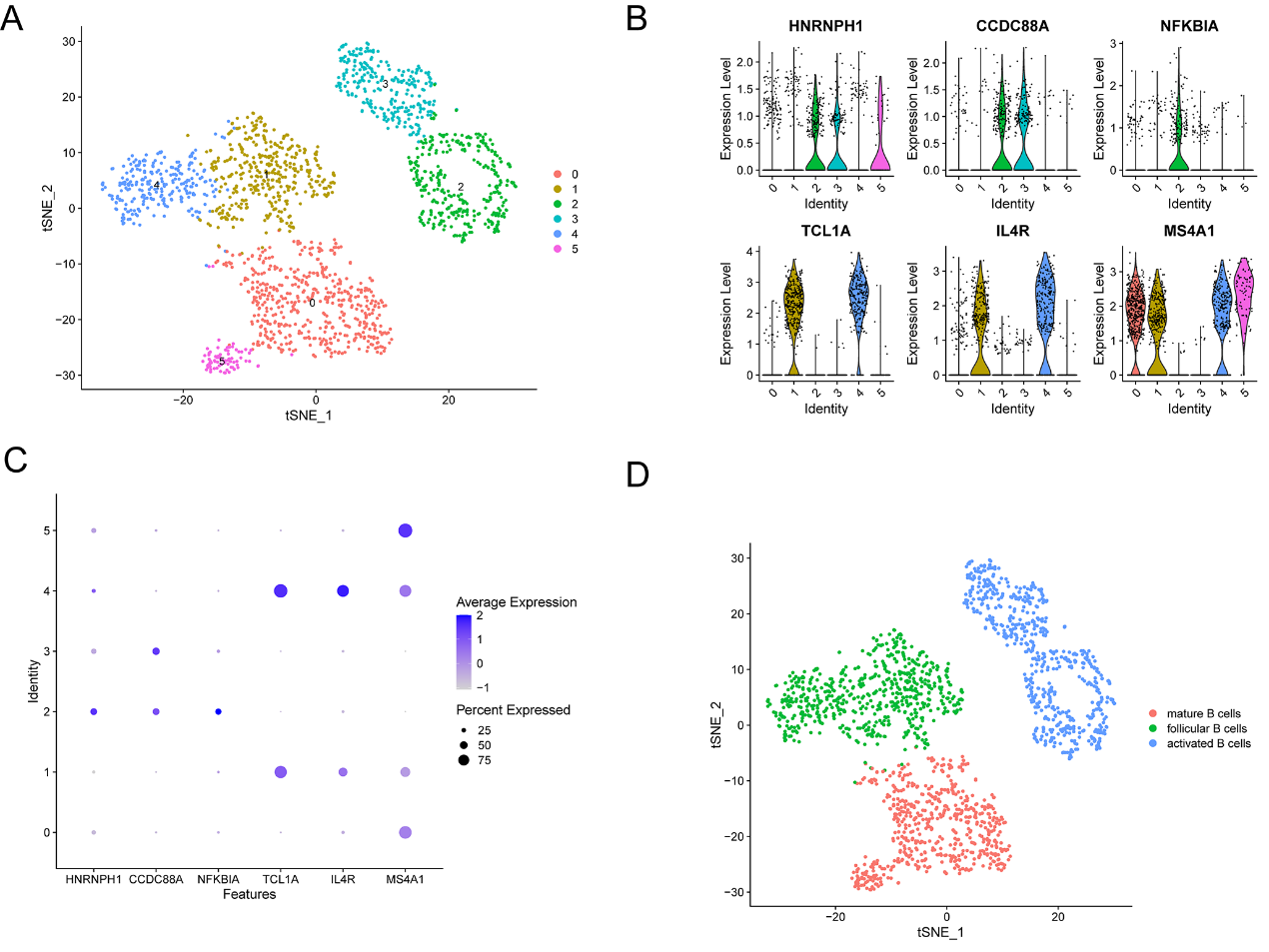


Figure S4 (A) Subtypes of B cells. (B) Violin plots of classical markers for B cells. (C) Dot plot of marker genes for distinct B cell types. (D) Three subtypes in B cells.


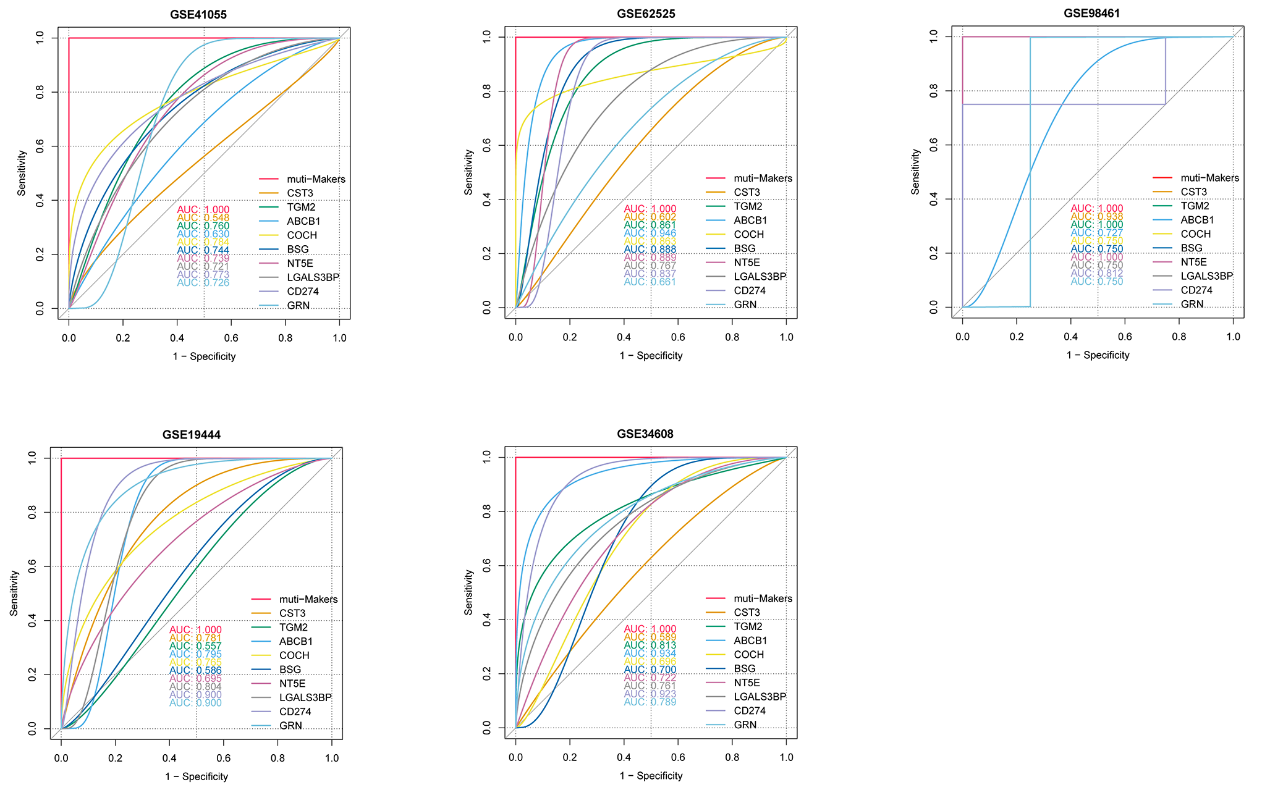


Figure S5 ROC curve of EVs-related hub genes in TB diagnosis in GSE41055, GSE62525, GSE98461, GSE19444, GSE34608 dataset.


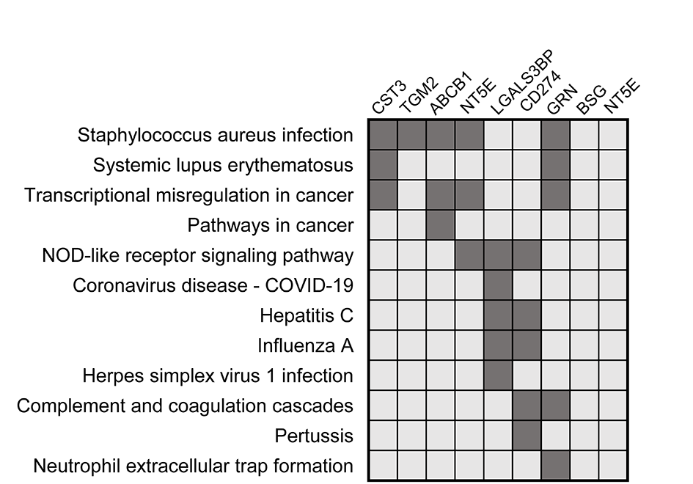


Figure S6 Heatmap displaying the GSEA investigation of EVs-related hub genes.

Table S1 Potential anti-TB small molecule compounds predicted by CMap analysis

|  | Mechenism of activities (moa) | Norm_cs | Drug status |
| --- | --- | --- | --- |
| pazopanib | VEGFR inhibitor\|KIT inhibitor\|PDGFR inhibitor | -1.9964 | Launched |
| baricitinib | JAK inhibitor | -1.9544 | Launched |
| BRD-K09991945 | PKC inhibitor | -1.9494 | Launched |
| pranlukast | Leukotriene receptor antagonist | -1.8486 | Unknown |
| masitinib | KIT inhibitor\|PDGFR inhibitor\|Src inhibitor | -1.8099 | Launched |
| losartan | Angiotensin receptor antagonist | -1.788 | Launched |
